# Antitumoral and Antimetastatic Activity by Mixed Chelate Copper(II) Compounds (Casiopeínas^®^) on Triple-Negative Breast Cancer, In Vitro and In Vivo Models

**DOI:** 10.3390/ijms25168803

**Published:** 2024-08-13

**Authors:** Mauricio M. González-Ballesteros, Luis Sánchez-Sánchez, Adrián Espinoza-Guillén, Jesús Espinal-Enríquez, Carmen Mejía, Enrique Hernández-Lemus, Lena Ruiz-Azuara

**Affiliations:** 1 Departamento de Química Inorgánica y Nuclear, Facultad de Química, Universidad Nacional Autónoma de México, Ciudad de México 04510, Mexico; mauricio.gb.117@outlook.com (M.M.G.-B.);; 2 Laboratorio de Biología Molecular del Cáncer, UMIEZ, Facultad de Estudios Superiores Zaragoza, Universidad Nacional Autónoma de México, Ciudad de México 09230, Mexico; 3 Departamento de Genómica Computacional, Instituto Nacional de Medicina Genómica, Ciudad de México 14610, Mexico; 4 Laboratorio de Biomedicina Interdisciplinaria, Facultad de Ciencias Naturales, Universidad Autónoma de Querétaro, Ciudad de México 76230, Mexico

**Keywords:** triple-negative breast cancer, metastasis, metallodrugs, copper, casiopeína

## Abstract

Triple-negative breast cancer (TNBC), accounting for 15–20% of all breast cancers, has one of the poorest prognoses and survival rates. Metastasis, a critical process in cancer progression, causes most cancer-related deaths, underscoring the need for alternative therapeutic approaches. This study explores the anti-migratory, anti-invasive, anti-tumoral, and antimetastatic effects of copper coordination compounds Casiopeína IIIia (CasIIIia) and Casiopeína IIgly (CasIIgly) on MDA-MB-231 and 4T1 breast carcinoma cell lines in vitro and in vivo. These emerging anticancer agents, mixed chelate copper(II) compounds, induce apoptosis by generating reactive oxygen species (ROS) and causing DNA damage. Whole-transcriptome analysis via gene expression arrays indicated that subtoxic concentrations of CasIIIia upregulate genes involved in metal response mechanisms. Casiopeínas^®^ reduced TNBC cell viability dose-dependently and more efficiently than Cisplatin. At subtoxic concentrations (IC_20_), they inhibited random and chemotactic migration of MDA-MB-231 and 4T1 cells by 50–60%, similar to Cisplatin, as confirmed by transcriptome analysis. In vivo, CasIIIia and Cisplatin significantly reduced tumor growth, volume, and weight in a syngeneic breast cancer model with 4T1 cells. Furthermore, both compounds significantly decreased metastatic foci in treated mice compared to controls. Thus, CasIIIia and CasIIgly are promising chemotherapeutic candidates against TNBC.

## 1. Introduction

Cancer is a term that defines a set of multifactorial diseases, where malignant cells possess various phenotypic abnormalities, such as loss of differentiation, a high proliferation rate, and malignant tumors that tend to grow invasively, destroying surrounding as well as distant normal tissues and organs [1,2]. These diseases are one of the main causes of morbidity and mortality worldwide; 10 million deaths were attributed in 2020. Particularly, female breast cancer is one of the most diagnosed cancers, with around 2.3 million new cases worldwide [3]. Importantly, approximately 15% of all breast cancers do not express estrogen receptor (ER), progesterone receptor (PR), or human epidermal growth factor receptor 2 (HER-2); this type is referred to as triple-negative breast cancer (TNBC). Specifically, most of these tumors have poorer survival compared to other breast cancer subtypes [4]. The biological behavior of TNBC is more aggressive, with early recurrences and a higher risk of death, in addition to presenting a greater tendency to develop distant metastases, compared to the other breast cancer subtypes, in which metastases in the liver, lung, bone, and central nervous system predominate [4,5]. In this regard, it has been described that the progression of cancer toward aggressive pathological grades with worse prognosis involves the processes of invasion and metastasis. These two events cause approximately 90% of cancer deaths since they allow malignant cells to spread, which hinders various aspects of the treatment of the disease [6,7,8,9].

Chemotherapy is the mainstay of treatment and generally involves the administration of anthracyclines, taxanes, and/or platinum compounds to alter the functions of cancer cells. In particular, Cisplatin, and also second- and third-generation platinum (carboplatin, oxaliplatin), remains among some of the most effective antitumor agents used in clinical practice [10]. Azim et al. (2019) give a current overview of the treatment of TNBC with a genomic approach, which highlights the use of combinatorial therapies with carboplatin, being very effective in TNBC with BRCA 1/2 mutations [4]. However, the clinical use of platinum-based drugs entails many serious side effects, such as nephrotoxicity, neurotoxicity, and myelosuppression. Due to this, the study of other metallodrugs with antineoplastic potential activity has been encouraged [11]. In this regard, the antitumor drugs based on endogenous metals (Co, Cu, Zn, and Fe) are less toxic than platinum analogues; moreover, by using these types of metals, synthesis and production costs could be reduced. Also, the pharmacological properties of metal complexes can be tuned by varying the nature of the ligand and donor atoms. Copper-containing coordination compounds are effective antitumor agents, and constitute a less expensive and safer alternative to classical platinum-containing chemotherapy [10]. 

**Figure 1:**
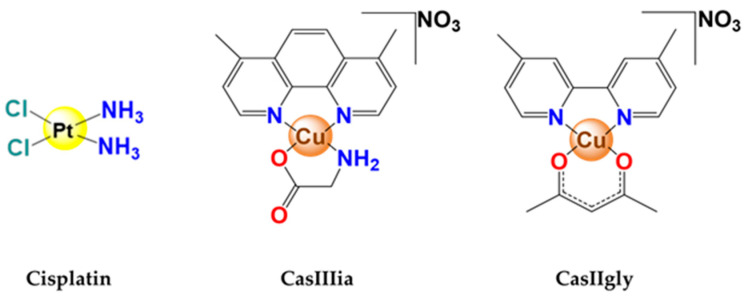
Structure of Cisplatin, Casiopeína IIgly (CasIIgly), and Casiopeína IIIia (CasIIIia).

The potential antitumor effect of Casiopeínas^®^ in different cancer models both in vivo and in vitro, in a wide variety of cell lines [12,13,14], are an alternative. Casiopeínas^®^ are a family of copper compounds with a bidentate ligand of the diimine (N-N) type such as phenanthroline or bipyridine and the second charged ligand of the N-O type (α-aminoacidate) or O-O donor (acetylacetonate or salicylaldehydate) (Figure 1). They are water, methanol, and glucose serum-soluble [15,16,17]. Regarding the mechanism of action, it has been shown that they can induce apoptosis [13,18,19] through an increase in ROS in mitochondria [20], DNA fragmentation, and activation of caspase-3; moreover, in the xenotransplant of colon cancer cell line HCT-15 on immunodeficient mice (Balb/C nu/nu), a greater reduction in tumor volume was observed compared to Cisplatin, correlating with an increase in apoptosis and a reduction in the mitotic index [13]. Furthermore, Casiopeínas^®^ are less toxic than Cisplatin due to their lower LD_50_ in different animal models. CasIIIia has been selected and approved to start clinical phase I studies [21,22]. 

Currently, several research groups have synthesized new copper(II) compounds, Casiopeínas^®^-like, which have shown cytotoxic and antiproliferative activity in different cancer cell lines, with ligands such as phenanthroline [23,24], doxycycline [25], and some flavonoids [26]. In addition, treatment with some of these compounds has shown anti-invasive activity in in vitro models, and they even decrease the expression of certain metalloproteases (MPP-2 and MPP-9) [23,24,25,26]. Similarly, a series of metallodrugs with some potential to inhibit migration, invasion, and metastasis processes has been described [27]; however, in contrast to Casiopeínas^®^, these compounds have low water solubility. Moreover, no studies have been performed on their mechanisms of action or toxicology, and studies of their biological activity using in vivo models are scarce. With respect to Casiopeínas^®^, there are few studies on their antimigratory or anti-invasive activity. Recently, Castillo-Rodriguez et al. (2021) reported that CasIIILa, having dose-dependent antiproliferative activity in different cancer cell lines such as cervical, prostate, breast, glioma, and colon, is also capable of reducing the invasive process by 60%, with subtoxic concentrations (IC_20_) in the glioma cell line U-373 MG [28]. Furthermore, there are studies describing the changes in gene expression induced by treatment with CasIIgly in the HeLa cell line, showing that it is able to decrease the expression of genes related to migration events such as AURKA, SNAIL2, BMP4, BMP6, and N-Cadherin [29,30,31]. 

To do this, we explored the antimigratory, anti-invasive, antitumoral, and antimetastatic effects of two different copper coordination compounds, CasIIIia and CasIIgly, on MDA-MB-231 and 4T1 breast carcinoma cell lines in vitro and in vivo, using subtoxic (IC_20_) and toxic concentrations (IC_50_). We also tested the effect of Cisplatin as a comparison. We developed a whole-transcriptome analysis via gene expression arrays (IC_20_) and then performed a differential gene expression analysis, as well as an over-representation analysis of those differential genes. The results altogether allowed us to discuss the effect of Casiopeínas^®^ on the migration and invasion of triple-negative breast cancer.

## 2. Results

In order to evaluate the effect of CasIIIia, CasIIgly, and Cisplatin on the viability of triple-negative breast cancer tumor cells, MDA-MB-231 and 4T1 cell cultures were treated with these compounds at different concentrations. Cell viability was evaluated by the MTT technique, and the concentration necessary to induce 20% (IC20) and 50% cell death (IC50) was determined (Table 1).

The calculation of IC_50_ and IC_20_ of metallodrugs (Casiopeínas^®^ and Cisplatin) in TNBC cell lines was performed using the dose-response model. 

The results obtained in Table 1 show that both CasIIIia and CasIIgly decreased the cell viability in a dose-dependent manner with respect to controls in the breast tumor cell lines. Also, these results indicate that on MDA-MB-231 and 4T1 cells, both CasIIIia and CasIIgly have better effects than Cisplatin, showing the same cytotoxic effect with a lower concentration. 

**Figure 2:**
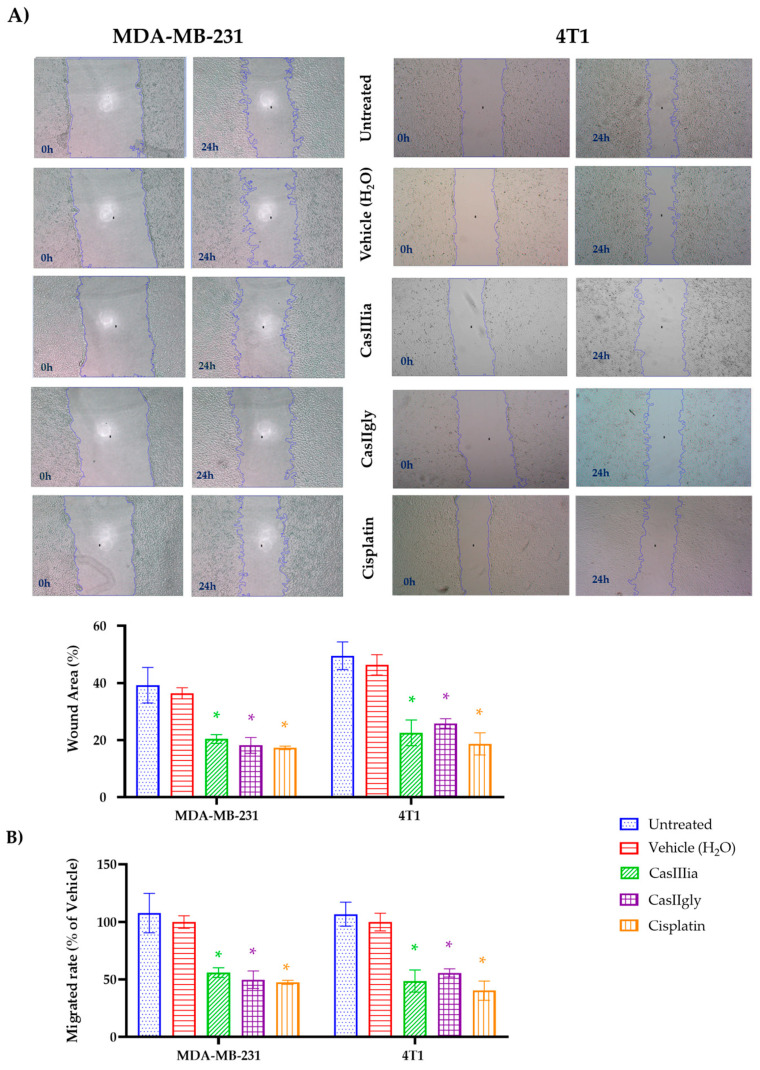
Metallodrugs inhibit migration of breast cancer cells: (**A**) Migration capacity of MDA-MB-231 and 4T1 cells was evaluated by wound-healing assay after treatment with metallodrugs (CasIIIia, CasIIgly, and Cisplatin) for 24 h. Representative photographs of wound-healing assay in the corresponding treatments from the initial time 0 h to the end of treatment 24 h, viewed at 40× are shown. (**B**) Analysis of wound area and percentage of migration with the respective treatments, in the MDA-MB-231 and 4T1 cells. Bars represent the means ± S.D. of at least three independent experiments. A one-factor ANOVA was performed using Tukey’s test for multiple comparisons. An asterisk (*) indicates statistically significant differences with respect to the untreated group and the vehicle group, with a significance of * *p* < 0.01. There were no significant differences between the CasIIIia, CasIIgly, and Cisplatin groups.

Once these results were obtained, the antimigratory capacity of CasIIIia and CasIIgly was determined, performing wound-healing assays in monolayer cultures of MDA-MB-231 and 4T1 cells, synchronized for 24 h by nutritional depletion. Subsequently, a wound was made in the monolayer and cell proliferation was inhibited with mitomycin C. Under these conditions, the cultures were treated with the corresponding IC_20_ for each metallodrug for 24 h, photographs were taken at time 0 and 24 h (Figure 2A). Finally, the area of the wounds in the different experimental conditions was determined with the Image J software Version 1.54 g, and the data were expressed as the percentage of cell migration.

The data obtained in Figure 2B indicate that CasIIIia, CasIIgly, and Cisplatin significantly inhibit the migration capacity of breast tumor cell lines MDA-MB-231 and 4T1. However, these cells have not only migratory capacity but also invasive capacity and migration for chemotaxis, so it is necessary to evaluate whether both CasIIIia and CasIIgly affect the invasive capacity of these tumor cell lines through chemo-migration.

**Figure 3:**
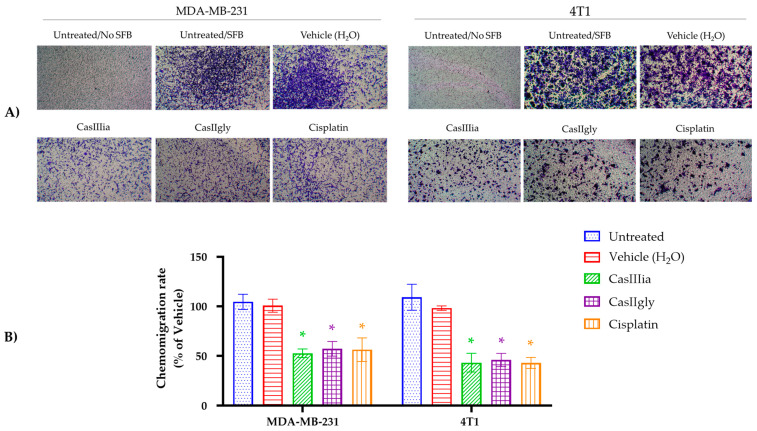
Metallodrugs inhibit chemo-migration of breast cancer cells. Chemo-migration capacity of MDA-MB-231 and 4T1 cells were assessed by transwell assay after exposure to metallodrugs (CasIIIia, CasIIgly, and Cisplatin) for 24 h: (**A**) Representative photographs of the chemo-migration assay in transwell chambers taken at 24 h with the different treatments (IC_20_). (**B**) The graphs represent the percentage of cells migrating at 24 h considering the vehicle group (H_2_O) as 100%, CasIIIia, CasIIgly, and Cisplatin. Each bar represents the mean ± S.D., n = 3. A one-factor ANOVA with Tukey’s test for multiple comparisons was performed, and the results were statistically significant. An asterisk (*) indicates statistically significant differences with respect to the untreated group and the vehicle group, with a significance of * *p* < 0.01. There were no significant differences between the CasIIIia, CasIIgly, and Cisplatin groups.

For this reason, a Boyden chamber assay was performed, where the ability of the cells to pass from one side of the chamber to the other was evaluated, showing not only the ability to migrate but also the ability to invade. MDA-MB-231 and 4T1 cells were put in a culture chamber and treated with CasIIIia, CasIIgly, and Cisplatin for 24 h, and photographs were taken of the cells that migrated by chemotaxis with each treatment (Figure 3A), and the number of cells that migrated to the other side were quantified and the data were expressed as a percentage of chemo-migration (Figure 3B). When comparing the statistical significance of the three metallodrugs, we observed that they are statistically significant against the control and vehicle but are not statistically significant among them.

The results showed that CasIIIia and CasIIgly inhibited the invasiveness of MDA-MB-231 and 4T1 breast cancer cells by approximately 50% for the applied concentrations (IC_20_). Therefore, we found that a concentration of these metallodrugs that decreases approximately 20% of cell viability in the wound-healing assay and transwell assay decreases the ability to migrate by approximately 50 to 60%. Taken together, these results suggest that CasIIIia and CasIIgly inhibit the migration and invasively cellular cells from tumoral cells, suggesting that these compounds have antimetastatic activity. Due to the previously mentioned background of CasIIIia, such as its lower toxicity, in contrast to Cisplatin, and its approval for clinical phase 1 studies, we decided to focus on it for antitumor and antimetastatic activity studies in the murine model of breast cancer (4T1) [21,22,32].

**Figure 4:**
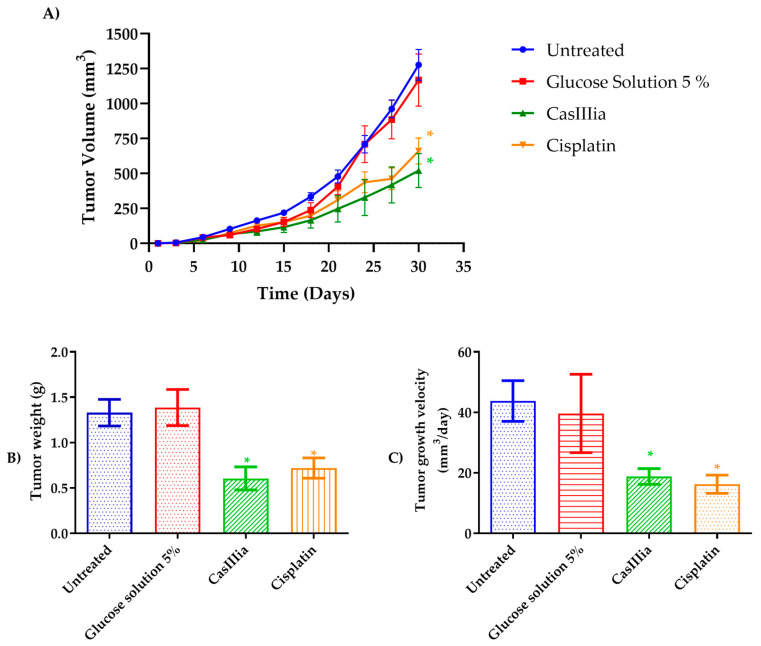
CasIIIia and Cisplatin inhibit tumor growth rate in a breast cancer mouse model (4T1 cells): (**A**) Tumor growth curve during the period of treatment. A one-factor ANOVA was performed with a Tukey’s test for multiple comparisons. The ANOVA was performed comparing all groups at day 30 (end of treatment). The tumor volume of the CasIIIia- and Cisplatin-treated groups was found to be statistically significant with respect to the untreated group and the glucose solution 5% group. (**B**) Tumor weight of each treated group at the end of the experiment. (**C**) The tumor growth velocity was calculated and the treatments with metallodrugs showed a significant effect. Each bar represents the mean ± S.E, n = 10. A one-factor ANOVA with Tukey’s test for multiple comparisons was performed and the results were statistically significant. An asterisk (*) indicates statistically significant differences with respect to the untreated group and the Glucose solution 5% group, with a significance of * *p* < 0.01. There were no significant differences between the CasIIIia, and Cisplatin groups.

**Figure 5:**
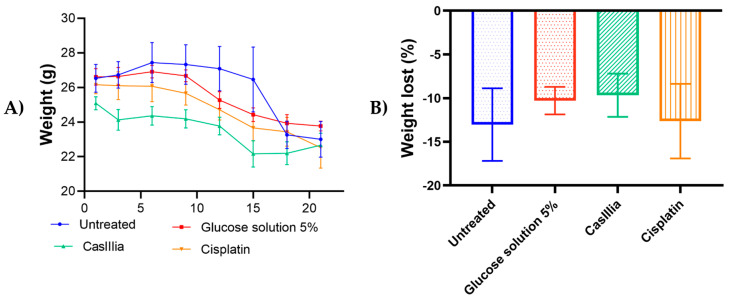
Monitoring and % Weight loss in Balb/c mice treated with Metallodrugs: (**A**) The initial and final weight of BALB/c mice during and after treatment. Mean body weight changes over 21 days after treatment. Each value represents a mean ± SE of n = 10. (**B**) Toxicity was measured by the percentage weight loss of the mice. The percentage weight loss, as an indicator of toxicity, was calculated for each animal as follows: [(weight on day 21/weight on day 0) − 1] × 100. Unpaired *t*-test was used to evaluate the statistical significance between groups.

**Figure 6:**
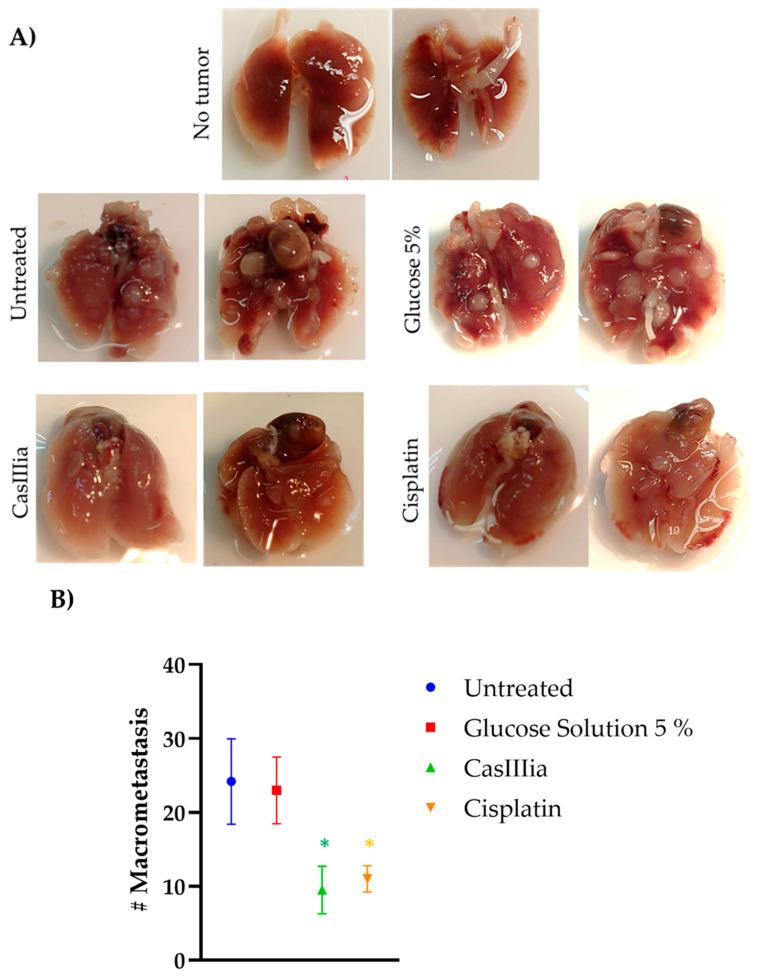
The in vivo antimetastatic effect of Metallodrugs: (**A**) Representative photographs of lungs of the different treatments. (**B**) Mean number of metastatic *foci* (Macrometastasis). Each point represents the mean ± S.E, n = 10. A Student’s *t*-test found that CasIIIia and Cisplatin were statistically different *t* with respect to the untreated group (the Student’s *t*-test was performed with standard error). An asterisk (*) indicates statistically significant differences with respect to the untreated group and the Glucose Solution 5% group, with a significance of * *p* < 0.01. There were no significant differences between the CasIIIia, and Cisplatin groups.

In order to confirm that CasIIIia has antitumoral and antimetastatic activity, in in vivo model BALB/c strain female mice were inoculated with 1 × 10^4^ cells of the 4T1 breast tumor cell line and treated with CasIIIia (6 mg/kg every 4 days, 6 doses), and Cisplatin (4 mg/kg every 7 days, 4 doses) for 30 days after the appearance of the primary tumor. The antitumoral activity was evaluated by growth tumor (volume mm^3^), tumor weight at the end, and the tumor growth velocity (Figure 4), as a toxicity indicator, weight monitoring was carried out and the % weight loss of the mice treated with metallodrugs was calculated. (Figure 5), while the antimetastatic activity was determined by the number of tumors observed on the lung (macrometastases) (Figure 6). 

The data obtained in Figure 4 established that CasIIIia and Cisplatin induced a decrease in volume tumoral of about 30%, with a lessening of tumoral weight in the order of 50%, and a lesser tumor growth velocity (60%), suggesting that CasIIIia and Cisplatin have similar antitumoral activity.

In addition, the weight of the mice at the end of the treatment was evaluated, where the trend was observed that only the groups treated with the 5% glucose solution and CasIII-ia recovered their post-treatment weight. However, the effect was not statistically significant with respect to the untreated group (Figure 5A), Toxicity was assessed using the percentage weight loss, as an indicator of toxicity (Figure 5B), which was calculated as follows: [(weight on day 21/weight on day 0) – 1] × 100 An unpaired Student’s *t*-test found that neither treated group had a statistically significant difference from the untreated group. Although the CasIII-ia group was not statistically significant, this group shows a trend of lower toxicity.

Figure 5B shows that there is a trend toward less weight loss with CasIII-ia than with Cisplatin. However, under these experimental conditions and treatment time, there was no significant difference between the treated and untreated groups.

The antimetastatic effect was evaluated at the end of the experiment, and the lungs were removed and evaluated by counting the number of macrometastases, finding that the groups treated with metallodrugs decreased by more than 50% of the number of macrometastases, in addition to showing a tendency to decrease the size of the macrometastases. With respect to antimetastatic activity from CasIIIia and Cisplatin, the results showed (Figure 6) that these compounds induced a lesser number of tumors in the lung regarding the controls; these results were statistically different, suggesting that CasIIIia and Cisplatin have antimetastatic activity.

Differential expression was performed independently for each drug, at three different concentrations: no treatment, sub-toxic concentration, and toxic concentration. 

Interestingly, the enriched biological processes show greater similarity when comparing different concentrations of the same compound rather than comparing different compounds. Specifically, the contrasts of Cisplatin, CasIIgly, and CasIIIia between IC_50_ and IC_20_ share more similarly enriched processes than those observed in the contrasts between CasIIIia_50_ vs. NT, and CasIIIia_20_ vs. NT (No treatment). This pattern holds consistently across all cases.

**Figure 7:**
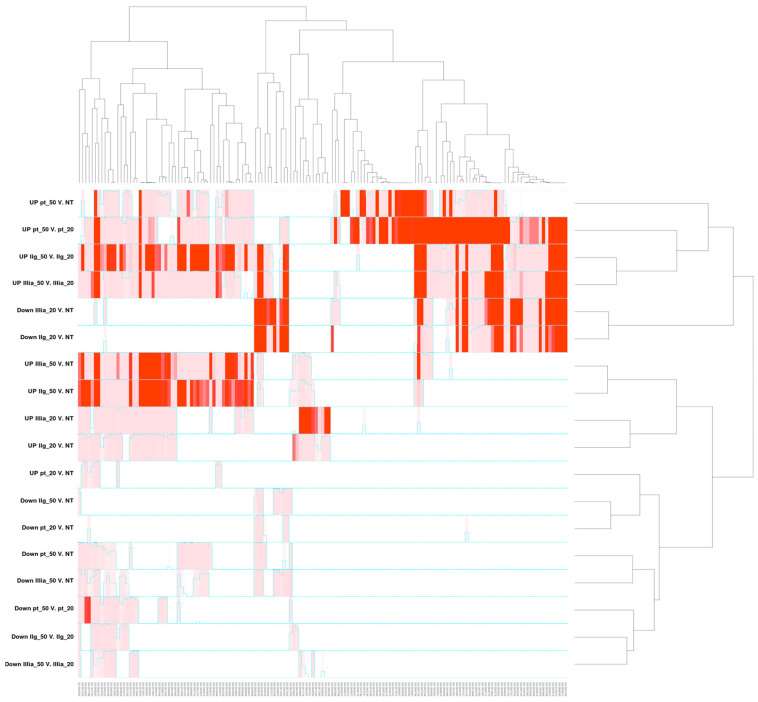
Heatmap of the enriched processes based on the differential expression analysis associated with each contrast (rows). Intensity color is proportional to the −log(*p*-val) of each process (for clarity, the list of enriched processes is provided in the Supplementary Material Table S1).

**Figure 8:**
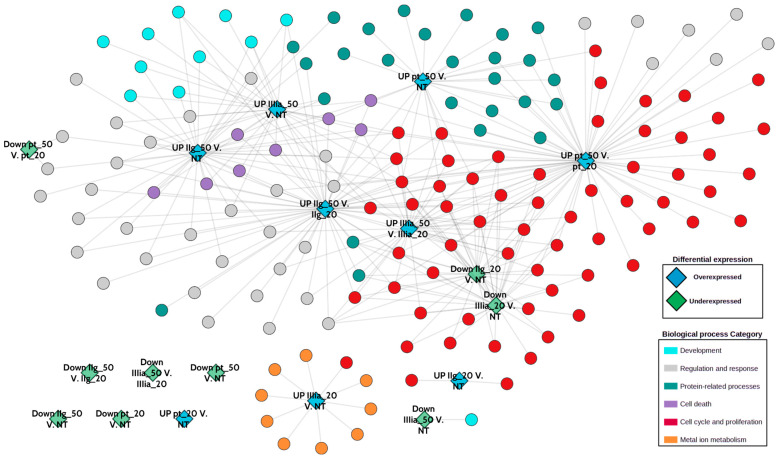
Network of enriched processes associated with the differential expression contrasts. Green diamonds represent underexpressed gene sets for the specific contrast. Blue diamonds take account of overexpressed gene sets. Circles show the biological processes linked to each contrast. Color of circles represents the global category of those processes. In Supplementary Material, we have provided the same figure with all the names of enriched biological processes.

In every instance, the IC_50_ vs. IC_20_ contrast exhibits the highest number of associated biological processes, surpassing even the contrasts of IC_50_ vs. NT and IC_20_ vs. NT. Figure 7 and Figure 8 illustrate this observation, where the most abundant biological processes in the heatmap and network are linked to the IC_50_ vs. IC_20_ contrasts (Figure 7), and the majority of nodes are directly linked to the IC_50_ vs. IC_20_ contrasts (Figure 8). Importantly, in Figure 8, we decided to separate the overexpressed gene sets (blue diamonds) from the underexpressed ones (green diamonds). 

As observed from Figure 8, the large majority of enriched biological processes belong to a small fraction of general processes: Cell cycle and proliferation, cell death, development, protein-related processes, regulation and response, and metal ion metabolism. From the figure, it is possible to appreciate that overexpressed gene sets present much more enriched processes (colored circles).

For instance, overexpressed genes in the contrasts with toxic concentrations (CasIIgly_50_, and CasIIIia_50_ vs. CasIIgly_50_ or NT) show a statistically significant association with processes related to development, as observed in the upper left part of the figure (turquoise circles). Overexpressed genes for other contrasts are strongly associated with cell cycle processes (red circles) or metabolic processes (dark green circles). 

An intriguing finding emerges from the contrasts of overexpressed genes in CasIIIia_20_ vs. NT: they are the only contrast showing biological processes associated with a metal ion response and metal metabolism (highlighted by the orange circles in the bottom left part of Figure 8). Such processes are absent in the differentially expressed genes when compared with toxic concentrations. Additionally, the contrast of overexpressed genes for CasIIgly_20_ vs. NT is the sole contrast exhibiting process related to hypoxia response.

Another notable observation is that only a few processes related to cell cycle, cell death, and protein assembly are significantly enriched in more than four contrasts. This suggests that only a limited number of processes are common to all compounds, despite the overall categories (represented by the colors of the circles) being nearly identical across all contrasts.

Lastly, it is worth noting that the majority of enriched processes are associated with overexpressed gene sets, except in the case of underexpressed gene sets of Casiopeínas^®^ at subtoxic concentrations compared to no treatment. In these cases, cell-cycle-related processes are equally enriched for both overexpressed and underexpressed gene sets. This indicates a more complex dynamic that warrants further in-depth analysis.

## 3. Discussion

Cisplatin plays a fundamental role in chemotherapy treatments, mainly because of its clinical efficacy directed against solid tumors such as ovarian, cervical, breast, esophageal, head and neck, and lung tumors; however, numerous side effects and drug resistance have been demonstrated [33,34,35]. Consequently, research on metallodrugs has intensively diversified, focusing particularly on those with a biometal center, and several investigations have focused on apoptotic and antiproliferative properties [33,36,37]. However, only a small number of researchers have looked at their alleged anti-invasive and antimetastatic properties. Breast cancer, one of the most prevalent malignancies and malignant tumors in women, is still severely hampered by metastatic spread, which is the primary cause of death from the disease [7,38,39]. In this regard, the Casiopeínas^®^ such as CasIIIia and CasIIgly have shown excellent antitumoral potential [12,13,19]; however, their antimetastatic activity has not been evaluated. Therefore, in this work, we evaluated the anti-invasive and antimetastatic activity of Casiopeínas^®^ in TNBC.

Hence, cell viability and IC_50_/IC_20_ values were determined. The results indicated that CasIIIia and CasIIgly have dose-dependent toxic effects on MDA-MB-231 and 4T1 cells, while Cisplatin requires a higher concentration to have the same cytotoxic effect as Casiopeínas^®^ (Table 1). These results are congruent with the previously reported antecedents of Casiopeínas^®^, which have shown genotoxic, cytotoxic, and antiproliferative activity in different types of cancer such as Ovarian, Cervical, Breast, Lung, Neuroblastoma, Sarcoma, Colon, Glioblastoma, etc. [12,14,18,20,40]. Furthermore, in different cell lines, Casiopeínas^®^ have shown lower cytotoxic activity than Cisplatin [12,41,42], and even in vivo models have shown better antitumor activity than Cisplatin by reducing the mitotic index and increasing the apoptotic index [13,19]. 

Recently this year, a great variety of metallodrugs with possible antimetastatic and anti-invasive activity has been described, which may interfere with or inhibit some mechanisms necessary for metastasis, such as motility, metalloprotease activity, epithelial–mesenchymal transition, angiogenesis, inflammation, and chemotaxis [27]. An example was reported by Stefano et al. (2022) of a new Pt (II)-complex containing 1,10-phenanthroline (phen), [Pt(η1-C2H4OMe)(DMSO)(phen)]Cl, with a decrease in cell migration/invasion in 2D and 3D in vitro models and decreasing MMP-9 and MMP-2 expression and activity in Neuroblastoma cells [43]. Another more specific example is that of a copper(II) with tropolone (trp) complex [Cu(trp)_2_], reported by Balsa, L. M. et al. (2020), their results showed that [Cu(trp)_2_] inhibited the cell migration and cell invasion of breast multicellular spheroids, and reduced mammosphere-forming capacity affecting the size and number of mammospheres [44].

In agreement with the aforementioned, our enrichment analysis of overexpressed genes in response to subtoxic concentrations of CasIIIia revealed a notable enrichment of genes associated with the cellular metal response pathway. This suggests a substantive upregulation of genes implicated in metal response mechanisms upon exposure to this concentration of the compound. Intriguingly, this heightened metal response gene expression is not observed in conjunction with another subset of differentially expressed genes across alternative experimental conditions, even those featuring toxic concentrations of CasIIIia. This nuanced observation may reflect the sophisticated regulatory modulation of these genes, rather than a dichotomous response solely tied to drug administration. Further investigations are warranted to elucidate the intricacies of this phenomenon.

Moreover, our analysis, as depicted in Figure 7 and Figure 8, underscores that a significant proportion of the identified differentially expressed genes play integral roles in cellular processes related to the cell cycle and metabolic pathways. This observation is consistent with the established pharmacological effects of these compounds, particularly in the context of influencing cancer cell cycle dynamics and metabolic regulation. Hence, the enrichment analysis presented herein accentuates the propensity of sub-toxic concentrations of CasIIIia to affect distinct cellular mechanisms governing metal response, distinguishing them from other concentration regimes.

It is worth noting that the majority of contrasts with associated enriched processes correspond to those of overexpressed genes. Taking into account that an enriched process contains a set of genes that allegedly participate in the said biological event, one can assume that the results shown in Figure 8 mostly refer to upregulated processes. If the latter is maintained as a working hypothesis, toxic concentrations of CasIIgly or CasIIIia may exert an increase in apoptosis-related pathways. Additionally, subtoxic concentrations of CasIIgly or CasIIIia clearly show a decrease in the cell cycle, as can be appreciated from Figure 8, where the green diamonds representing underexpressed genes in the contrasts of CasIIgly_20_ or CasIIIia_20_ vs. NT are surrounded by cell cycle processes.

Concomitant with the latter, toxic concentrations of Cisplatin may increase cell metabolism (dark green circles in the upper part of Figure 8) and, at the same time, exacerbate the cell cycle. 

These examples should be carefully analyzed, since over-representation analyses such as the one presented here do not take into account the activator/inhibitory role of a particular gene in a pathway. Further investigation is necessary to assess these results. However, it is important to highlight the trends observed after this comprehensive genomic analysis.

The relevance of differential expression and over-representation analyses in anti-cancer drug investigation is paramount. These analyses allow for the identification of key genes and pathways that are affected by drug treatments, providing insights into the molecular mechanisms of drug action and resistance. For instance, studies have shown that understanding the differential expression of genes can lead to the identification of biomarkers for drug response and resistance [45,46]. Furthermore, over-representation analysis can highlight specific biological processes and pathways that are disproportionately affected, offering potential targets for therapeutic intervention [47].

This kind of detailed molecular insight is crucial for the development of more effective anti-cancer therapies. By understanding how drugs like Casiopeínas^®^ modulate specific pathways and processes at sub-toxic concentrations, researchers can design strategies to enhance therapeutic efficacy while minimizing side effects. Such approaches are fundamental to precision medicine, where treatments are tailored to the unique genetic and molecular profile of each patient’s tumor [48].

In this regard, migration by wound-healing assay demonstrated the capability of CasIIIia and CasIIgly for inhibition of the migration of TNBC cell lines (Figure 2), and the results clearly demonstrate the Casiopeínas^®^ are similar to Cisplatin for the inhibition of migration with subtoxic concentrations (IC_20_). Subsequent assays by the transwell chamber elucidated the suppressive effects of CasIIIia and CasIIgly on the mobility and invasion of MDA-MB-231 and 4T1 cells. Again, Casiopeínas^®^ and Cisplatin showed comparable anti-mobility and anti-invasion effects with subtoxic concentrations (Figure 3). It should be noted that Castillo-Rodríguez et al. (2021) have demonstrated the dose-dependent inhibition of migration and invasion of U373 human glioma cells by a member of the family of Casiopeínas^®^ (CasIIILa) with subtoxic concentrations (IC_30_ e IC_20_) [28]. The proposed mechanism by which CasIIILa exerts an antiproliferative effect, promoting apoptotic cell death and inactivating the invasive process, is due to the generation of ROS, inactivating GSK3β, activating JNK and ERK, and promoting nuclear accumulation of β-catenin [28].

In this sense, CasIIIia and CasIIgly could be acting in a similar way, although future studies will be required to confirm this. Another possibility is that it is working in a similar way to Cisplatin, as reported by Whang et al. (2021), whereby Cisplatin inhibits cancer metastasis through blocking early steps of EMT. It antagonizes TGF-β signaling through suppressing the transcription of many genes involved in cytoskeleton reorganization and filopodia formation, which occur early in EMT and are responsible for cancer metastasis. Mechanistically, TGF-β and fibronectin-1 (FN1) constitute a positive reciprocal regulation loop that is critical for activating TGFβ/SMAD3 signaling, which is repressed by Cisplatin-induced expression of ATF3 [49]. In this context, it has been reported that CasIIgly decreases the gene expression induced by treatment with CasIIgly in the HeLa cell line, showing that it is able to decrease the expression of genes related to migration events such as TGF-β receptor type-1, SNAIL2, BMP4, BMP6, and N-Cadherin [29,30,31]. With these results, we suggest that CasIIIia and CasIIgly may work similarly to CasIIILa due to the background of the Casiopeína^®^ family, since many members of the family share mechanisms of action such as inductors of apoptosis, DNA fragmentation, generation of ROS, and cell cycle arrest. However, in order to discern this, the objectives of the present study were expanded to determine the mechanisms of migration reduction in breast cancer cells (MAD-MB-231 and 4T1), evaluating global gene expression changes by microarrays and presence variations in proteins related to motility and mesenchymal–epithelial transition by Western blot assays; these results will be finalized and published soon. 

A syngeneic murine xenograft model with the 4T1 cell line was used to evaluate the antitumor and antimetastatic effect. We administered CasIIIia and Cisplatin as reference drugs; we found that both treatments significantly decreased the volume, weight, and growth rate of the primary tumor in contrast to the untreated groups. However, there were no significant differences between the CasIIIia and Cisplatin groups (Figure 4). In addition, significant differences were found in the number of macrometastases in the lung compared to the untreated groups (Figure 5). This is in agreement with that reported by Whang et al. (2021), where Cisplatin in this same 4T1 model decreased tumor growth and the number of macrometastases in the lung [49]. On the other hand, the results for the case of CasIIIia are in agreement with previous experiments where an antitumor activity similar to Cisplatin is observed. Thus, as reported by Carvallo-Chaigneau et al. (2008), which describes that in an in vivo model of colon cancer, CasIIIia and CasIIgly decrease the relative volume of the tumor, in a similar way and with even greater efficiency than Cisplatin [13]. In addition, recently Aguilar-Jiménez et al. (2022) described similar results in the murine breast cancer model with the 4T1 cell line, using a new nano-encapsulated formulation with niosomes and CasIIIia, showing antitumor activity similar to Cisplatin but with lower toxicity [32].

Metastasis represents one of the main causes of mortality in cancer cases and, in the particular case of breast cancer, it is vitally important to achieve effective treatments for TNBC. Because these types of tumors are highly aggressive in the vast majority of cases, it is important to note that the activity of Casiopeínas^®^ has not previously been evaluated in TNBC cell lines, and therefore, together with the background of Casiopeínas^®^ and this new evidence on the potential to reduce cell migration, chemotaxis, and the number and size of metastases, to reaffirm the great antitumor potential of Casiopeínas^®^ in different types of cancer as good therapeutic candidates against TNBC. 

## 4. Materials and Methods

### 4.1. Cell Culture

MDA-MB-231 and 4T1, highly invasive breast carcinoma cell lines, and HeLa, adenocarcinoma of a cervix cell line, were purchased from the American Type Culture Collection (ATCC, Rockville, MD, USA) and cultured in RPMI-1640 medium (GIBCO, Carlsbad, CA, USA) containing 10% heat-inactivated Fetal Bovine Serum (FBS, GIBCO, Carlsbad, CA, USA) with phenol red, supplemented with benzylpenicillin. Cultures were maintained in a humidified atmosphere with 5% CO_2_, at 37 °C. All cell-based assays were performed using cells in the exponential growth phase. 

### 4.2. Metallodrugs

The copper(II) complex’s CasIII-ia (CAS [223930–33–4]) and CasIIgly were synthesized at the National Autonomous University of Mexico according to the specifications outlined in the corresponding patent [15,16,17]. Cisplatin (cis-Diamineplatinum(II) dichloride) was procured from Sigma-Aldrich (Mexico City, Mexico) (Catalog number # 479306-1G).

### 4.3. Viability Determination by MTT

Cell viability was measured by the conventional MTT reduction assay as previously described and developed by Mosmann (1983) [50,51]. Briefly, HeLa, MDA-MB-231, and 4T1 cells were inoculated at a density of 1 × 10^5^ cells/well in 96-well plates for 24 h in 100 µL of RPMI-1640 with 10% FBS. Following seeding, the culture supernatant was removed, and refresh medium RPMI-1640 with 10% FBS containing various concentrations of Cisplatin, CasIIIia, and CasIIgly were added, and the cells were incubated for 24 h. H_2_O was used to dissolve the metallodrugs previously mentioned. Also, 1% of vehicle H_2_O was added as a control. MTT dye (20 µL, 5 mg/mL) was added to each well and the plate was incubated for 4 h at 37 °C in a CO_2_ incubator. The formed formazan crystals were solubilized by adding 100 µL of isopropanol/sodium dodecyl sulfate (SDS)/1M HCl mixture [52,53,54]. The absorbance of MTT-formazan was measured using a microplate reader (AgilentReader™, ACTGene, Piscataway, NJ, USA) at 560 nm.

### 4.4. Determination of IC_50_ and IC_20_

Cytotoxicity activity is expressed as the amount of the compound required to inhibit the viability of cells by 50% (IC_50_—Inhibitory concentration 50%) or 20% (IC_20_—Inhibitory concentration 20%). The calculated value was obtained by means of the dose-response model using data analysis software (Origin 2020).

### 4.5. Wound-Healing Assay

Cells were seeded 3 × 10^5^ cells per well, in 24-well plates, to 90% confluence in 500 µL of 10% SFB RPMI-1640 medium and allowed to adhere for 24 h at 37 °C and 5% CO_2_. Subsequently, the supernatant was removed and washed 3 times with 250 µL of PBS supplemented with Ca^2+^/Mg^+2^, and 700 µL of RPMI-1640 medium without serum was added and left for at least 16 h (4T1) and up to 18 or 24 h (MDA-MB-231), in order to remove proliferation signals and synchronize the cells. Subsequently, scraping was performed in a single direction as close to the center of the plate from top to bottom, without exerting much force, and then 3 washes were performed with RPMI-1640 medium at 1% SFB to remove as many suspended cells as possible and to add survival signals. The treatments with the compounds were placed in quadruplicate and prepared in RPMI-1640 medium at 1% SFB. Experimental groups were Control: “Untreated”, only the culture medium was changed to fresh medium; Vehicle control: Water and Treatments: CasIIIia (IC_20_), CasIIgly (IC_20_), and Cisplatin (IC_20_). Subsequently, photographs were taken of the cells at time 0 h and 24 h, making sure to capture the same quadrant. The images were processed with ImageJ software, using the “Wound_Healing_Tool” Plugin, and the change in the area occupied by the cells was calculated from time 0 to 24 h [55,56].

### 4.6. Transwell Assay (Chemotaxis Migration Assay)

For the chemotaxis migration assay, the QCM 24-well colorimetric cell migration assay (ECM508; Chemicon, Temecula, CA, USA), was used. Assay steps were performed according to the manufacturer’s protocol. Briefly, for the migration assay, MDA-MB-231 and 4T1 cells that had been stabilized by passage two to three times were harvested using Trypsin-ethylenediaminetetraacetic acid solution (GIBCO, Carlsbad, CA, USA). Harvested cells were grown to 70% confluence in six-well plates, washed twice with PBS, and incubated for another 18 h in serum-free medium for cell starvation. The cells were then incubated and were detached, counted, and dissolved in a serum-free medium. Cells (2 × 10^5^) were brought into the upper chamber, containing various concentrations (IC_20_) of Cisplatin, CasIIIia, and CasIIgly, which was then inserted into the lower chamber with 500 µL of medium with 10% fetal bovine serum. Each chamber was incubated for 24 h at 37 °C in 5% CO_2_. After removing medium from the top side by gentle pipetting, the upper chamber was placed into a clean well containing 400 µL of cell-staining solution and incubated for 20 min at room temperature to stain migrated cells. The upper chamber was rinsed and cleaned with water to remove non-migrated cells and air-dried according to the manufacturer’s instructions. The stained upper chambers were then transferred to clean wells containing 200 µL of extraction buffer and incubated for 15 min at room temperature. The optical density at 560 nm was then measured by transferring the extraction buffer (100 µL) to a 96-well microtiter plate [57].

### 4.7. In Vivo 4T1 Model, Determination of Antitumor and Antimetastatic Activity

For each experiment, a total of 40 healthy female mice (6–8 weeks old) of the BALB/c strain were maintained in the Bioterium of the FES-Zaragoza, UNAM, under controlled conditions of temperature (22 °C), a relative humidity of 50–60%, and 12 h light–dark cycles, with lights on between 7:00 and 21:00 h. 

A total of 1 × 10^4^ viable 4T1 mammary adenocarcinoma cells of no greater than 5 pass in a volume of 100 µL of serum-free RPMI were inoculated into the right mammary gland of 5–6-week female BALB/c mice. Tumors were allowed to develop and grow for approximately 32 days. Tumor area was monitored by measuring the largest diameter × smallest diameter of the tumor using a digital vernier (Fowler sylvac) and the approximate volume was calculated by (a × b^2^) × π/6, where a and b are the largest and smallest dimensions of the tumor. 

After the appearance of the tumor (about 10 days after implantation), treatments were administered intraperitoneally (ip). The animals were divided into 4 groups, distributed as follows: Untreated; Glucose solution 5% 6 doses every 4 days; CasIIIia (6 mg/kg) 6 doses every 4 days; Cisplatin (4 mg/kg) 4 doses every 7 days. At day 32 post-implantation (end of treatment), mice were sacrificed, and organs such as lung, liver, kidneys, ovary, and primary tumor were removed and fixed with 4% paraformaldehyde (Sigma-Aldrich, Mexico City, Mexico), and subsequently the number of macrometastases in the lungs was counted [58]. 

All animals were handled according to the Guide for the Care and Use of Laboratory Animals of the National Institutes of Health and the National Regulation for the Care and Use of Experimental Animals (NOM-062-ZOO-1999). All experimental protocols were approved by the Ethics Committee of FES-Zaragoza, with the approval number: FEZ-CE/22-118-08.

### 4.8. RNA Extraction and Preparation

RNA was extracted from the MDA-MB-231 cells (5 × 10^6^ cells) without treatment, and those cells treated with metallodrugs (Cisplatin, CasIIIia, and CasIIgly) in different concentrations (subtoxic “IC_20_” and toxic “IC_50_”) for 6 h, with the RNeasy Micro Kit (QIAGEN Mexico City, Mexico, Cat. No. 74004), according to the manufacturer’s instructions. RNA and nucleic acid concentration were measured with a spectrophotometer (Amersham Pharmacia Biotech, Barneveld, The Netherlands). Reverse transcription was performed with Superscript II Reverse Transcriptase (Invitrogen, No. cat.18064-014) and cDNA was amplified with Taq DNA Polymerase (Invitrogen, Mexico City, Mexico, No. cat. 11615-010). PolyA+ RNA from MDA-MB-231 cells were selected by affinity chromatography using an oligo (dT) cellulose column.

### 4.9. Differential Expression Analysis

Differential gene expression was conducted by comparing each one of the three different metallodrugs (Cisplatin, CasIIIia, and CasIIgly) between the different concentrations (subtoxic “IC_20_” and toxic” IC_50_”) for 4 h, and without treatment. Therefore, we conducted nine comparisons. Differential expression was performed with the R package DeSeq-2 [59]. For these analyses, we considered a |log2-FC| > 1 and a *p*-value < 0.01. Table 2 shows the comparisons.

In all cases, we separated the overexpressed genes and underexpressed genes to perform the functional enrichment analysis for each contrast. 

Functional enrichment analysis was performed by using an over-representation tool G-profileR [60]. We used the gene ontology biological process categories. In a nutshell, the list of differentially expressed genes is tested against the genes included in the GO categories. A hyper-geometric test is performed to quantify the statistical significance of the match size between the two lists. For this analysis, we decided to be severe in terms of statistical significance. Hence, we kept only those categories with an enrichment *p*-value < 1 × 10^−5^.

Afterward, gene ontology categories were grouped into more general categories to observe the global response of the treatments in terms of the associated process for each contrast.

### 4.10. Statistical Analysis

All experiments were performed in a triplicate of three independent experiments. In graphics for in vitro assays, bars represent the means ± standard deviation (S.D.) of at least three independent experiments. Data from experiments were analyzed by one-factor ANOVA, followed by a Tukey multiple comparison test. *p* < 0.05 was regarded as significant. 

For in vivo assays, the bar represents the mean ± standard error (S.E), n = 6, of three independent experiments. Data from experiments were analyzed by one factor ANOVA, followed by a Student’s *t*-test comparison test with Glucose solution 5%. *p* < 0.05 was regarded as significant.

## 5. Conclusions

In summary, our in vitro study demonstrates that Casiopeínas^®^ decrease the viability of TNBC cells in a dose-dependent manner and with greater efficiency than Cisplatin, in addition, at subtoxic concentrations (IC_20_), they decrease random migration and migration by chemotaxis of MDA-MB-231 and 4T1 cells, in a percentage similar to Cisplatin (approximately 50 to 60%). While in the in vivo murine breast cancer model, CasIIIia and Cisplatin significantly and similarly decrease the tumor growth velocity, the volume, and the final weight of the primary tumor. Relevant clues regarding the molecular mechanisms involved were further examined via whole-transcriptome gene expression analysis, in particular those related to the cell response to metal ions at subtoxic concentrations of CasIIIia, as well as an important increment in differentially expressed genes related to apoptosis, cell cycle, and metabolism. Importantly, these processes are exacerbated in the contrasts of CasIIIia and CasIIgly, but not in Cisplatin contrasts. Furthermore, it is important to note that treatment with CasIIIia and Cisplatin significantly decreased the number of metastatic *foci* in the mice treated, compared to the control groups. 

Therefore, our results indicate that CasIIIia is a good candidate as a chemotherapeutic agent against triple-negative breast cancer, as is CasIIgly.

## Figures and Tables

**Table 1:** Metallodrugs reduce the viability of breast cancer cells.

	Cell Line	MDA-MB-231		4T1	
Metallodrug		IC_50_ (µM)	IC_20_ (µM)	IC_50_ (µM)	IC_20_ (µM)
CasIIIia		19.56 ± 1.9	12.35 ±0.63	11.99 ± 0.73	9.72 ± 0.79
CasIIgly		1.27 ± 0.07	1.16 ± 0.04	0.88 ± 0.06	0.65 ± 0.01
Cisplatin		23.44 ± 1.5	12.67 ± 1.8	14.65 ± 1.1	9.95 ± 0.9

Data represent the mean ± SD for three independent experiments.

**Table 2:** Comparison table of the differential expressions of metallodrug treatments.

Cisplatin	CasIIgly	CasIIIia
IC_50_ vs. IC_20_	IC_50_ vs. IC_20_	IC_50_ vs. IC_20_
IC_50_ vs. NT	IC_50_ vs. NT	IC_50_ vs. NT
IC_20_ vs. NT	IC_20_ vs. NT	IC_20_ vs. NT

## Data Availability

Data are contained within the article and Supplementary Material.

## Supplementary Materials

The following supporting information can be downloaded at: https://www.mdpi.com/article/10.3390/ijms25168803/s1.
