# Supplementary material for: Antitumoral and Antimetastatic Activity by Mixed Chelate Copper(II) Compounds (Casiopeínas®) on Triple-Negative Breast Cancer, In Vitro and In Vivo Models
Source: Int J Mol Sci. 2024 Aug 13;25(16):8803. doi: 10.3390/ijms25168803 (PMC11354730; doi:10.3390/ijms25168803)
Supplement: Supplementary file 1 [file ijms-25-08803-s001.zip › ijms-3115912-supplementary.pdf]

**Table S1. List of the enriched processes for all contrasts. P-value  e-10**

| | |
|---|---|
| GO:0000070 | mitotic sister chromatid segregation |
| GO:0000075 | cell cycle checkpoint signaling |
| GO:0000077 | DNA damage checkpoint signaling |
| GO:0000079 | regulation of cyclin-dependent protein serine/threonine kinase activity |
| GO:0000086 | G2/M transition of mitotic cell cycle |
| GO:0000122 | negative regulation of transcription by RNA polymerase II |
| GO:0000226 | microtubule cytoskeleton organization |
| GO:0000278 | mitotic cell cycle |
| GO:0000280 | nuclear division |
| GO:0000470 | maturation of LSU-rRNA |
| GO:0000819 | sister chromatid segregation |
| GO:0001568 | blood vessel development |
| GO:0001666 | response to hypoxia |
| GO:0001890 | placenta development |
| GO:0001892 | embryonic placenta development |
| GO:0001933 | negative regulation of protein phosphorylation |
| GO:0001944 | vasculature development |
| GO:0002237 | response to molecule of bacterial origin |
| GO:0006259 | DNA metabolic process |
| GO:0006260 | DNA replication |
| GO:0006261 | DNA-templated DNA replication |
| GO:0006275 | regulation of DNA replication |
| GO:0006281 | DNA repair |
| GO:0006325 | chromatin organization |
| GO:0006334 | nucleosome assembly |
| GO:0006338 | chromatin remodeling |
| GO:0006364 | rRNA processing |
| GO:0006457 | protein folding |
| GO:0006882 | cellular zinc ion homeostasis |
| GO:0006915 | apoptotic process |
| GO:0006974 | cellular response to DNA damage stimulus |
| GO:0006986 | response to unfolded protein |
| GO:0007017 | microtubule-based process |
| GO:0007049 | cell cycle |
| GO:0007051 | spindle organization |
| GO:0007052 | mitotic spindle organization |
| GO:0007059 | chromosome segregation |
| GO:0007088 | regulation of mitotic nuclear division |
| GO:0007093 | mitotic cell cycle checkpoint signaling |
| GO:0007098 | centrosome cycle |
| GO:0007346 | regulation of mitotic cell cycle |
| GO:0008283 | cell population proliferation |
| GO:0008284 | positive regulation of cell population proliferation |
| GO:0008608 | attachment of spindle microtubules to kinetochore |
| GO:0009266 | response to temperature stimulus |
| GO:0009408 | response to heat |

Enriched processes

| | |
|---|---|
| GO:0009628 | response to abiotic stimulus |
| GO:0009890 | negative regulation of biosynthetic process |
| GO:0010273 | detoxification of copper ion |
| GO:0010389 | regulation of G2/M transition of mitotic cell cycle |
| GO:0010558 | negative regulation of macromolecule biosynthetic process |
| GO:0010563 | negative regulation of phosphorus metabolic process |
| GO:0010564 | regulation of cell cycle process |
| GO:0010648 | negative regulation of cell communication |
| GO:0010941 | regulation of cell death |
| GO:0010942 | positive regulation of cell death |
| GO:0010948 | negative regulation of cell cycle process |
| GO:0012501 | programmed cell death |
| GO:0016072 | rRNA metabolic process |
| GO:0022402 | cell cycle process |
| GO:0023057 | negative regulation of signaling |
| GO:0030490 | maturation of SSU-rRNA |
| GO:0031327 | negative regulation of cellular biosynthetic process |
| GO:0031570 | DNA integrity checkpoint signaling |
| GO:0032200 | telomere organization |
| GO:0032496 | response to lipopolysaccharide |
| GO:0033046 | negative regulation of sister chromatid segregation |
| GO:0033048 | negative regulation of mitotic sister chromatid segregation |
| GO:0033554 | cellular response to stress |
| GO:0033673 | negative regulation of kinase activity |
| GO:0033993 | response to lipid |
| GO:0034470 | ncRNA processing |
| GO:0034501 | protein localization to kinetochore |
| GO:0034605 | cellular response to heat |
| GO:0034660 | ncRNA metabolic process |
| GO:0034728 | nucleosome organization |
| GO:0035966 | response to topologically incorrect protein |
| GO:0042026 | protein refolding |
| GO:0042127 | regulation of cell population proliferation |
| GO:0042254 | ribosome biogenesis |
| GO:0042273 | ribosomal large subunit biogenesis |
| GO:0042274 | ribosomal small subunit biogenesis |
| GO:0042325 | regulation of phosphorylation |
| GO:0042326 | negative regulation of phosphorylation |
| GO:0042592 | homeostatic process |
| GO:0042770 | signal transduction in response to DNA damage |
| GO:0042981 | regulation of apoptotic process |
| GO:0043065 | positive regulation of apoptotic process |
| GO:0043067 | regulation of programmed cell death |
| GO:0043068 | positive regulation of programmed cell death |
| GO:0044770 | cell cycle phase transition |
| GO:0044772 | mitotic cell cycle phase transition |
| GO:0044786 | cell cycle DNA replication |

## Enriched processes

| | |
|---|---|
| GO:0044839 | cell cycle G2/M phase transition |
| GO:0045444 | fat cell differentiation |
| GO:0045597 | positive regulation of cell differentiation |
| GO:0045653 | negative regulation of megakaryocyte differentiation |
| GO:0045786 | negative regulation of cell cycle |
| GO:0045787 | positive regulation of cell cycle |
| GO:0045892 | negative regulation of DNA-templated transcription |
| GO:0045930 | negative regulation of mitotic cell cycle |
| GO:0045934 | negative regulation of nucleobase-containing compound metabolic process |
| GO:0045936 | negative regulation of phosphate metabolic process |
| GO:0046686 | response to cadmium ion |
| GO:0048285 | organelle fission |
| GO:0048514 | blood vessel morphogenesis |
| GO:0048646 | anatomical structure formation involved in morphogenesis |
| GO:0051052 | regulation of DNA metabolic process |
| GO:0051093 | negative regulation of developmental process |
| GO:0051253 | negative regulation of RNA metabolic process |
| GO:0051254 | positive regulation of RNA metabolic process |
| GO:0051276 | chromosome organization |
| GO:0051301 | cell division |
| GO:0051348 | negative regulation of transferase activity |
| GO:0051726 | regulation of cell cycle |
| GO:0051783 | regulation of nuclear division |
| GO:0051784 | negative regulation of nuclear division |
| GO:0051985 | negative regulation of chromosome segregation |
| GO:0061644 | protein localization to CENP-A containing chromatin |
| GO:0061687 | detoxification of inorganic compound |
| GO:0065004 | protein-DNA complex assembly |
| GO:0070482 | response to oxygen levels |
| GO:0071276 | cellular response to cadmium ion |
| GO:0071280 | cellular response to copper ion |
| GO:0071294 | cellular response to zinc ion |
| GO:0071310 | cellular response to organic substance |
| GO:0071396 | cellular response to lipid |
| GO:0071459 | protein localization to chromosome, centromeric region |
| GO:0071824 | protein-DNA complex subunit organization |
| GO:0072331 | signal transduction by p53 class mediator |
| GO:0072359 | circulatory system development |
| GO:0090068 | positive regulation of cell cycle process |
| GO:0090329 | regulation of DNA-templated DNA replication |
| GO:0097501 | stress response to metal ion |
| GO:0098813 | nuclear chromosome segregation |
| GO:0140014 | mitotic nuclear division |
| GO:1901700 | response to oxygen-containing compound |
| GO:1901796 | regulation of signal transduction by p53 class mediator |
| GO:1901987 | regulation of cell cycle phase transition |
| GO:1901988 | negative regulation of cell cycle phase transition |

Enriched processes

| | |
|---|---|
| GO:1901990 | regulation of mitotic cell cycle phase transition |
| GO:1901991 | negative regulation of mitotic cell cycle phase transition |
| GO:1902679 | negative regulation of RNA biosynthetic process |
| GO:1902749 | regulation of cell cycle G2/M phase transition |
| GO:1902850 | microtubule cytoskeleton organization involved in mitosis |
| GO:1903047 | mitotic cell cycle process |
| GO:1903083 | protein localization to condensed chromosome |
| GO:1903507 | negative regulation of nucleic acid-templated transcription |
| GO:1904029 | regulation of cyclin-dependent protein kinase activity |
| GO:1905819 | negative regulation of chromosome separation |
| GO:1990169 | stress response to copper ion |
| GO:2000026 | regulation of multicellular organismal development |
| GO:2000816 | negative regulation of mitotic sister chromatid separation |

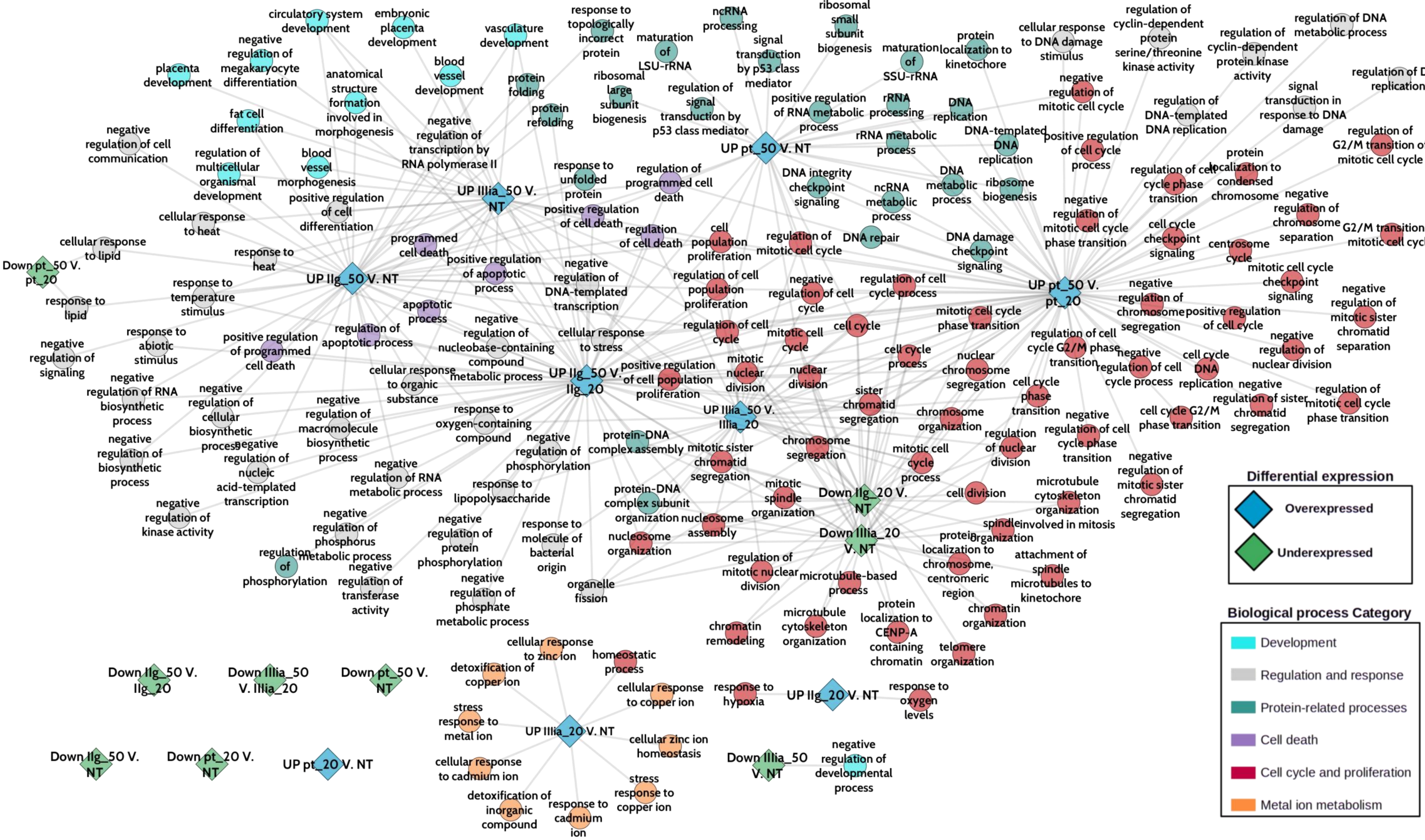


**Figure S1. Network of enriched processes associated with the differential expression contrasts.**
